# The relationship between serum 25-hydroxy vitamin D status and hypertension in Syrian population: retrospective cohort study

**DOI:** 10.1097/MS9.0000000000001989

**Published:** 2024-03-25

**Authors:** Nouha Abd AL-Hameid Bakkar, Aliaa Youssef Bakr, Ayham Haitham Alhusseini, Zaynab Haidar Alourfi

**Affiliations:** Departments ofa Endocrinology; b Oncology; c Neurology, Damascus University, Faculty of Medicine, Damascus, Syria

**Keywords:** 25-hydroxyvitamin D, hypertension, nutrition, Syria

## Abstract

**Introduction:**

Vitamin D is a liposoluble steroid hormone that plays a crucial role in the maintenance of bone metabolism and calcium homoeostasis. Many studies on the effects of vitamin D on general health have been significantly increased, driven by new findings concerning the systemic and extraskeletal effects of this hormone. This study was performed to determine whether low levels of vitamin D were associated with hypertension in Syrian people.

**Materials and methods:**

This retrospective cohort study consisted of 207 subjects, including 83 (40.1%) patients suffering from essential hypertension and 124 (59.9%) patients with normal blood pressure. Aged older than 18 years, who was referred to the endocrinology clinic from September 2022 to September 2023. The data were analysed by using SPSS (version 25). Logistic regression analyses were performed with adjustments for age, sex, and waist circumference.

**Results:**

Hypertension rates were 73%, 20%, and 5% in 25-hydroxyvitamin D groups less than 12 ng/ml, 12–20 ng/mL, and greater than or equal to 20 ng/ml, respectively. Odds ratios (95% CIs) for hypertension adjusting for age, sex, and waist circumference were 178.6 (30.5_1045.6), 5.13 (0.9_26.5) for 25-hydroxyvitamin D levels less than 12 ng/ml, and 12–20 ng/ml, respectively, compared with the greater than or equal to 20 ng/ml group.

**Conclusions:**

This study has shown a high prevalence of low vitamin D levels (25OHVD/20 ng/ml) among a sample of Syrian people (78.3%). The lowest 25OHVD group was associated with a higher prevalence of hypertension, which refers to an adverse association between vitamin D level and essential hypertension. Further research is needed to confirm this relationship.

## Introduction

Highlights

- Low vitamin D levels have been associated with essential hypertension.
- High prevalence of vitamin D deficiency among the Syrian population.
- Females had lower vitamin D levels than males.

Vitamin D is a liposoluble steroid hormone that plays a crucial role in the maintenance of bone metabolism and calcium homoeostasis^1^. Humans obtain vitamin D from ultraviolet B (UVB) radiation and from diet or supplementation in the case of vitamin D deficiency. The skin is known as the major source of vitamin D^2^. Vitamin D deficiency is common worldwide. There is still no unified value that defines vitamin D deficiency. Different cut-off values have been set by the Endocrine Society (ES) and the Institute of Medicine (IOM), whereby desirable 25OHVD levels were set at 30 ng/ml versus 20 ng/ml, respectively^3,4^. Current guidelines suggest that 25-hydroxyvitamin D values less than 12 ng/ml are associated with an increased risk of rickets/osteomalacia^5^.

During the last few years, many studies have shown that vitamin D acts on many extraskeletal targets. Thus, low levels of vitamin D may contribute to the occurrence of chronic diseases^6^. Vitamin D receptors (VDRs)are expressed in heart tissue and can be modulators of cardiovascular diseases, including hypertension^7^.

Hypertension is one of the most important risk factors for cardiovascular disease, which is the major cause of morbidity and mortality worldwide^8^. More than one billion adults worldwide have hypertension^9^. Uncontrolled hypertension has been associated with complications including atherosclerosis, cardiomyopathy, cerebrovascular events, and renal failure^10^. Hypertension is due to specific causes in a small fraction of cases, but in the vast majority of individuals (>90% of cases), its aetiology cannot be determined, and this is called essential hypertension^11^.

The mechanism by which vitamin D may regulate blood pressure is not firmly established. One of the possible mechanisms is the putative role of 1, 25(OH)2D as a negative regulator of the renin-angiotensin system (RAS)^12^.

This study aimed to determine any correlation between vitamin D and hypertension.

## Methods

### Setting and sampling

This retrospective cohort study consisted of 207 subjects. All subjects were selected from the outpatient endocrinology clinic over a period from September 2022 to September 2023.

The study protocol was approved by the Research Ethics Committee of Damascus University and in accordance with the Declaration of Helsinki and in line with the STROCSS criteria)^13^.

The inclusion criteria were as follows: primary hypertension and age older than 18 years. Exclusion criteria: patients with secondary hypertension, pregnant women, diabetic patients, any disease or drug that affects vitamin D absorption and metabolism, and patients receiving vitamin D or calcium treatments.

The sample size of 207 was calculated by this formula:


n=N*X/(X+N–1),where,X=Zα/22−*p*(1−p)/MOE2,

Zα/2 is the critical value of the Normal distribution at α/2 (e.g. for a confidence level of 95%, α is 0.05 and the critical value is 1.96), MOE is the margin of error 5%, p is the sample proportion, and N is the population. It was obtained by random sampling.

### Tools and planning

A complete clinical history and physical examination of all patients were performed by the same doctor to evaluate the exclusion criteria. Waist circumference was measured midway between the lower rib margin and the iliac crest following a normal expiration. All patients underwent the following biochemical measurements: creatinine, liver function tests, calcium, albumin, fasting blood glucose (after overnight fasting for at least 8 h), and serum 25-hydroxyvitamin D (25OHVD). Other tests were performed based on clinical suspicion.

25OHVD levels were measured using an automated electrochemiluminescence immunoassay (Elecsys 2010 analysers, Roche Diagnostic Gmbh, Mannheim, Germany). A serum PTH assay was performed in the same laboratory using the chemiluminescence method (DPC 2000,Siemens, Germany).

Arterial blood pressure was measured twice after 5 minutes of rest with a mercury sphygmomanometer on the arm at the heart level of the seated subject. Hypertension was defined as systolic blood pressure greater than or equal to 140 mmHg and diastolic blood pressure greater than or equal to 90 mmHg^14^ and/or a prior diagnosis of hypertension or current use of antihypertensive drugs irrespective of blood pressure.

Plasma 25OHD concentrations were divided into three categories according to the Institute of Medicine (IOM) classification: less than 12 ng/ml, 12–20 ng/ml, greater than or equal to 20 ng/ml^15^. These divisions were chosen because the cut-off value that defines vitamin D deficiency is close to that in our country, according to a previous study in Damascus city^16^.

### Statistical analysis

SPSS (version 25) was used to analyse the data. Counts and percentages were used to summarise categorical variables, and the mean and standard deviation were used for continuous measures. We used nonparametric statistics such as χ^2^ test and parametric statistics such as analysis of variance (ANOVA) and *P* value less than 0.05 was considered statistically significant. Logistic regression analysis was used to estimate odds ratios (ORs) with 95% CIs for the association between 25OHD levels and the presence of hypertension. We adjusted for potential confounding by age, sex, and waist circumference. The crude and adjusted ORs for hypertension were calculated for each 25OHD category. The category with 25OHD levels greater than or equal to 20 ng/ml was used as the reference group.

## Results

**Figure 1:**
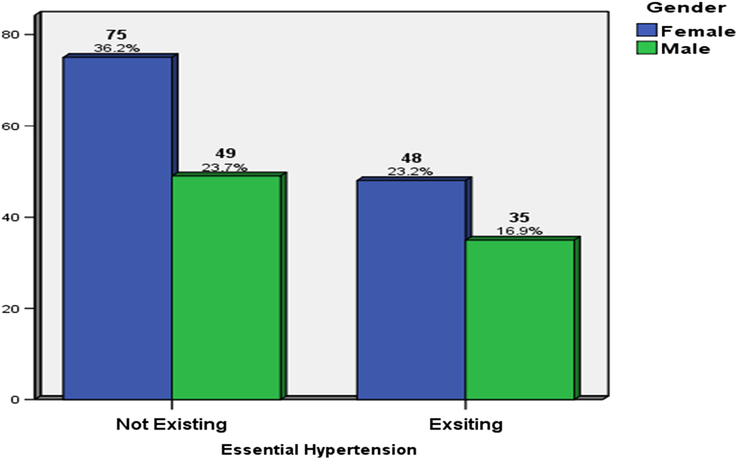
Relative distribution of patients according to sex and hypertension in the total study sample.

**Figure 2:**
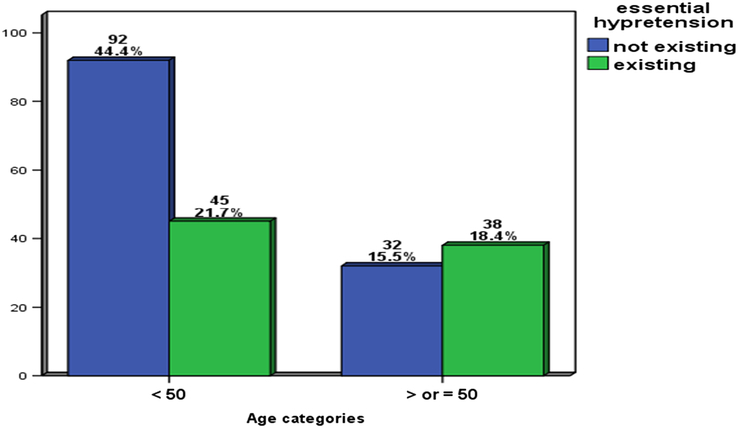
Relative distribution of patients according to age and hypertension in the total study sample.

Patients' characteristics are presented in Table 1. Of 207 patients, 84 (40.6%) were male, and 123 (59.4%) were female with mean age (45.3±11.3). Mean serum 25OHD was (14.5±9.1). The overall prevalence of hypertension in the study population was 40.1%. The prevalence of hypertension was higher among ages older than or equal to 50 years (*P*<0.003) (Fig. 1). Hypertension existence did not show a significant difference between females and males (*P*>0.703) (Fig. 2).

**Table 1:** Characteristics of the study participants

Statistics								
Variable	Number	Mean	Median	Mode	Std. deviation	Range	Min	Max
Age	207	45.3	45	48	11.3	58	18	76
25OHVD (ng/ml)	207	14.5	13	9^a^	9.1	50.7	2.5	53.2
PTH (pg/ml)	88	115.18	98.55	40	62.66	202	30	232
Ca (Corr) (mg/dl)	207	9	9	9	0.5	2.5	7.5	10
FBG (mg/dl)	207	82.7	83	81^a^	7.6	34	64	98
Waist circumference (cm)	207	88.62	90	91	7.54	40	65	105

a Multiple modes exist—The smallest value is shown.

25OHVD, 25 hydroxyl vitamin D; Corr, corrected calcium; FBG, fasting blood glucose; PTH, parathyroid hormone.

Characteristics of participants stratified by 25OHVD groups are shown in Table 2.

**Table 2:** Characteristics of participants according to 25OHVD groups

25OHVD (ng/ml)	<12 *N*=92	12–20 *N*=70	≥20 *N*=45	*P*
V.D, mean±SD	7.31±2.54	15.26±2.16	28.11±8.47	<0.0001
Age, mean±SD	46.49±10.56	45.16±12.37	42.33±10.80	0.130
Male, *n* (%)	27 (29.3)	31 (44.3)	26 (57.8)	0.005
Female, *n* (%)	65 (70.7)	39 (55.7)	19 (42.2)	
Waist circumference, mean±SD	87.41±7.20	89.89±7.58	89.11±7.93	0.043
PTH, mean±SD	179.68±23.87	84.06±18.84	44.43±6.89	<0.0001

25OHVD, 25 hydroxyl vitamin D; PTH, parathyroid hormone.

Serum 25OHVD levels less than 12 ng/ml were found in 92 subjects (44.4%), serum 25OHVD levels of 12–20 ng/ml in 70 subjects ( 33.8% ), and serum 25OHVD levels greater than or equal to 20 ng/ml in 45 subjects (21.7% ). Age had no statistically significant difference between groups. Females had significantly lower 25OHVD levels than males. Patients in the 12–20 ng/ml 25OHVD group had the highest median waist circumference. There was a statistically significant inverse correlation observed between 25OHD and PTH levels.

**Figure 3:**
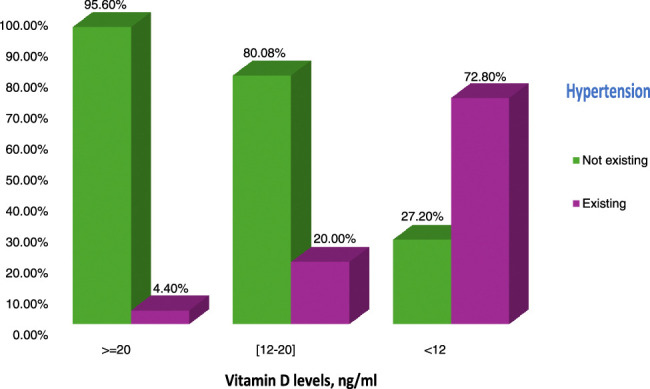
Rate of hypertension within 25OHVD groups.

Patients in the lowest 25OHVD group had the highest prevalence rate of hypertension (*P*<0.001) (Fig. 3)

The association between plasma 25OHVD concentration and hypertension is shown in Table 3.

**Table 3:** Associations (ORs with 95% CI) of 25OHVD and the presence of hypertension

	Hypertension			
	Existing	Not existing	Odds ratio (95% CI)	
	*n* (%)	*n* (%)	Crude OR (95% CI)	adjusted OR (95% CI)
25OHVD categories				
<12 ng/ml	67 (72.8)	25 (27.2)	57.62 (12.98–155.7)	178.68 (30.53–1045.6)
12–20 ng/ml	14 (20)	56 (80)	5.38 (1.16–24.92)	5.13 (0.99–26.55)
≥20 ng/ml	2 (4.4)	43 (95.6)	1.0 (reference)	1.0 (reference)

25OHVD, 25 hydroxyl vitamin D.

The crude OR for hypertension was 57.62 (95% CI: 12.98–155.75 ) and 5.38 (95% CI: 1.16–24.92) ) for 25OHVD groups less than 12 ng/ml and 12–20 ng/ml, respectively, compared with 25OHVD group greater than or equal to 20 ng /ml. In the multivariate analyses, adjusting for covariates of age, sex, and waist circumference, the OR remained statistically significant throughout the groups (Table 3).

## Discussion

This study showed a high prevalence of low vitamin D levels (25OHVD˂20 ng/ml) among a sample of Syrian people (78.3%). We attributed these results to poor nutrition and consumption of a small amount of foods rich in vitamin D, such as fatty fish and cod liver. These results were close to the results of a previous study in Syria that was conducted in Damascus and its surroundings between April 2011 and March 2013, which showed higher vitamin D levels in men than women and 90.1% of the population had 25OHVD levels less than 20 ng/ml^17^.

These results are consistent with the high rate of low vitamin D levels worldwide, especially in Middle Eastern countries. The rates of 25OHVD levels less than 20 ng/ml were 81%, 64%, and71.9% in Saudi Arabia, Qatar, and Lebanon, respectively^18,19,20^. This may suggest that the specific cut-off value of vitamin D deficiency is different in Middle Eastern countries.

In our study, females had lower vitamin D levels than males. This difference may be due to wearing the hijab and skin-covering clothes and spending more time at home than males. A high prevalence of vitamin D deficiency among females was also observed in a study in Jordan, and Saudi Arabia^21,22^. Age had no effect on vitamin D levels in our study.

Vitamin D plays a key role in regulating blood pressure in several ways. In addition to potential effects on the renin-angiotensin system and regulation of vascular smooth muscle contractility, vitamin D has also been hypothesised to have other effects on vascular endothelium and smooth muscle. It can induce the activation of vasodilatory and antithrombotic genes^23^. Moreover, vitamin D seems to suppress inflammation and atherosclerotic parameters and thus provides vascular protection^24^. Additionally, the role of hyperparathyroidism in cardiovascular diseases and the ability of 1,25(OH)2 D to inhibit parathyroid hormone (PTH) and hyperparathyroidism are being considered^25,26^.

The results of our study showed that the greatest rate of hypertension was at 25OHVD levels less than 12 ng/ml. The lowest 25OHVD group was associated with a higher prevalence of hypertension (OR 57.62, 95% CI: 12.98–155.7). This reflects a significant inverse association between low vitamin D levels and hypertension. These results were consistent with the results of a cross-sectional study that included 4125 subjects. This epidemiological study showed an inverse relation between serum 25OHD levels and blood pressure, but it did not find serum 25OHD levels to be a significant predictor of future hypertension^27^. Similar results were observed in another cross-sectional study, which evaluated 2722 individuals. The hypertension rates were 52.4%, 40.8%, 27.2%, and 19.4% for 25OHD levels less than 15 ng/ml, 15–29 ng/mL, 30–39 ng/ml, and greater than or equal to 40 ng/ml, respectively^28^.

Conflicting results were observed in a longitudinal Aging Study in Amsterdam that included 1205 participants. This study concluded that 25OHD levels did not have any significant relation with high blood pressure, in contrast to PTH levels, which are associated with high blood pressure, possibly due to the relatively high levels of 25OHD in the study sample^29^. The last study also conflicts with a cross-sectional study that was conducted on 1420 Chinese participants, which concluded that serum 25OHD and PTH levels are not independently associated with the risk of hypertension^30^.

Interventions with vitamin D supplementation on blood pressure have been described. In a pilot feasibility study Judd *et al.*^31^ have demonstrated that blood pressure could be reduced with active form of vitamin D (calcitriol). Conversely, meta-analyses of vitamin D supplementation have reported weak evidence to support an effect on lower blood pressure^32^. More interventional studies are needed to determine the effect of vitamin D compounds on blood pressure.

The strength of our study is that all diagnoses were based only on confirmed hypertension. Additionally, the models were adjusted for potentially important confounders, including age, sex, and waist circumference. However, we cannot entirely rule out the possibility that the associations we observed were partly attributed to residual confounding.

Limitations of our study include the lack of data on other confounders such as, smoking status and physical activity, which were not controlled in our analyses due to insufficient data for the entire study population. Additionally, there were limited materials for testing PTH and 25OHD levels in our hospital laboratories. Also, our study population did not distribute evenly across 25OHD groups, and thus, relative comparisons were not entirely accurate.

## Conclusion

Our study observed higher rates of hypertension in patients with low 25OHD levels. Therefore, an optimal level of 25OHD concentrations is thought to be crucial for normal blood pressure. Interventional studies are needed to determine whether optimisation of 25OHD levels may help to prevent or ameliorate hypertension.

## Ethical approval

This study complies with the guidelines for human studies and was conducted ethically in accordance with the World Medical Association Declaration of Helsinki.. This study protocol was reviewed and approved by the Ethics Committee of the institution. Ethical approval for this study (Ethical Committee N° 3611) was provided by the Medical Research Ethics Council (MTCE) in Damascus University, Damascus, Syria on 30 August 2022.

## Consent

Written informed consent was obtained from the patient for publication and any accompanying images. A copy of the written consent is available for review by the Editor-in-Chief of this journal on request.

## Source of funding sources

Damascus University- funder No.501100020595 funds this research.

## Author contributions

N.B. contributed to design of the work, analysis and interpretation of data for the work and reviewing it critically for important intellectual content and Final approval of the version to be published. A.B. contributed to analysis and interpretation of data for the work and reviewing it critically for important intellectual content and final approval of the version to be published. AA reviewed the manuscript critically for important intellectual content and final approval of the version to be published and Z.A. contributed to design of the work and reviewed it critically for important intellectual content and final approval of the version to be published.

## Conflicts of interest disclosure

The authors have no conflicts of interest to declare.

## Research registration unique identifying number (UIN)

1. Research used: Research Registry www.researchregistry.com
2. UIN 9780.
3. Thelinkhttps://www.researchregistry.com/browse-the-registry#home/.

## Guarantor

Dr Nouha Bakkar.

## Data availability statement

All necessary details are available in the article. Further enquiries can be directed to the corresponding author.

## Provenance and peer review

Not commissioned, externally peer-reviewed.
